# A clinician's guide to omics resources in dermatology

**DOI:** 10.1111/ced.15117

**Published:** 2022-03-03

**Authors:** Brent J. Doolan, John A. McGrath, Alexandros Onoufriadis

**Affiliations:** ^1^ St John's Institute of Dermatology School of Basic and Medical Biosciences King's College London London UK

## Abstract

With recent advances in high‐throughput technologies, we are now in an era where the use of large‐scale datasets of biological samples and individual diseases can be analysed using omics methodologies. These include genomics, transcriptomics, proteomics, metabolomics, lipidomics and epigenomics. Omics approaches have been developed to deliver a holistic understanding of systems biology, to identify key biomarkers, and to aid in the interpretation of molecular, biochemical and environmental interactions. Navigating through the plethora of online datasets to find useful and concise information for comparison of data can be complex and overwhelming. The purpose of this article is to review the current repositories and databases, and to evaluate their application in dermatological research and their relevance to clinical practice. For this study, an extensive review of online platforms used in dermatology research was undertaken. Online resources for genetic disease information, genetic disease connection platforms for patients and researchers, clinical interpretation of variants, genome and DNA databases, and omics data repositories and resources were collected. This study provides a comprehensive overview of relevant databases that will aid clinicians and scientists using omics data in dermatology.

## Introduction

Traditionally, clinicians have relied on an in‐depth understanding of a patient's medical history and clinical examination to make an accurate diagnosis and to establish an appropriate treatment plan. However, in the 21st century there has been a great advance in scientific technologies, primarily spearheaded by innovations in genetics.^1^ Decreases in DNA sequencing costs and increases in the speed and accuracy of reading DNA have moved genetics and quantitative genetic data into mainstream dermatology.^1^ We are now in an age where the global scientific community has accumulated vast amounts of publicly available data, including clinical, epidemiological and molecular information.

Collectively, these advances provide a platform allowing dermatology datasets to be analysed for prospective improvements in diagnostics, therapeutics and insights into dermatological diseases. Specifically, data obtained from patients, biological samples or a particular disease can be compared with those from other datasets generated via high‐throughput analyses of omics approaches, such as genomics (the study of gene expression changes at a genome‐wide level), transcriptomics (the study of the complete set of RNA transcripts that are produced by the genome), proteomics (the study of dynamic protein products and their interactions), metabolomics (the study of cellular metabolites and their processes), lipidomics (the study of the structure and function of lipids) and epigenomics (the study of how cells control gene activity without changing the DNA sequence).^1^

These multiomics approaches have been developed in an attempt to provide precision medicine for patients, while also endeavouring to deliver a holistic understanding of a ‘systems biology’ and an interpretation of genotype–phenotype interactions.^2^ From a clinical setting, the challenge in using omics data is to generate concise, meaningful information, without being overwhelmed by the complexity of the data. Navigating through this process can be challenging, with > 700 web resources providing access to many thousands of systems (for example, http://pathguide.org/). In this review, we navigate through the tools and repositories that provide accurate dermatology datasets and explain how their clinical applications can be used in dermatology.

## Resources

### Genomics

There have been huge advances in next‐generation sequencing (NGS) technologies, which have led to data generation for genomes (single nucleotide polymorphisms, copy number variants and rare variants).^2^ Because of the benefits of diagnostics and reduced costs, the use of genetic testing is becoming more widely used in diagnostic laboratories, with many physicians integrating genomics into their management plans.^1^

To accurately interpret genetic findings, there has been substantial investment into establishing genetic disease databases, with web‐based sites such as Online Mendelian Inheritance in Man^3^ and OrphaNet^4^ constructed to maintain updated information on published skin diseases and their associated gene mutations (Table 1). The Genetic and Rare Diseases Center,^5^ Orphanet^4^ and GeneReviews^6^ offer clinicians an overview of diseases, as well as providing lay information for patients on their disease, where to find expert centres for management of genetic diseases and information on genetic counselling.

**Table 1:** Comparison of the available online resources for clinical genomics data for skin disease.

Online resource	Functionality	Source	References^a^
Genetic disease information			
OMIM	A continuously updated catalogue of human genes and genetic disorders and traits, with particular focus on the relationship between genetic variation and phenotypic expression. OMIM entries include a full text summary of each genetic phenotype and links to DNA and protein sequence, PubMed references, mutation databases and gene nomenclature	https://www.omim.org	Amberger *et al*., 2019
OrphaNet	A resource for information on rare diseases, which also contains a database of orphan drugs for rare diseases, as well as locations of expert centres for each disease. They report functional limitations of each disease and emergency guidelines for rare diseases	https://www.orpha.net	Weinreich *et al*., 2008
GeneReviews	A point‐of‐care resource for clinicians providing clinically relevant and medically actionable information for inherited conditions. Provides clinical guidelines for establishing the diagnosis, along with the clinical description, required molecular testing, management and genetic counselling for each disease. Contains 804 disease chapters	https://www.ncbi.nlm.nih.gov/books/NBK1116/	Adam *et a*l., 2010
GARD	Contains information on more than 6500 rare and/or genetic diseases, with information on current research studies and clinical trials of rare diseases, genetic testing resources, custom literature reviews and information in lay language for patients	https://rarediseases.info.nih.gov	Lewis *et al*., 2017
Genetic disease connection platforms for patients and researchers			
MyGene2	Enables searches of candidate variants/genes linked to phenotypic profiles of persons and families deposited in the Repository for Mendelian Disorders Family Portal. Users of MyGene2 can search for candidate variants matching a gene, inheritance model and/or phenotypic trait or profile. It has the ability to track variants of unknown significance among other families. Requires registration	http://www.mygene2.org	Chong *et al*., 2016
GeneMatcher	Enables connections between clinicians and researchers who share an interest in the same gene(s). When a match occurs, each submitter receives an automatic email notification. Further communication is at the discretion of the submitters. GeneMatcher also has an option to match on OMIM phenotype numbers and genomic position	http://www.genematcher.org	Sobreira *et al*., 2015
DECIPHER	An interactive web‐based database that provides a genome browser for gene searching including sequence variants, as well as providing a human phenotype ontology search engine, where phenotype features can be selected to find possible gene mutations, linked to ~30 000 patients with phenotypic abnormalities	https://www.deciphergenomics.org/	Firth *et al*., 2009
Café Variome	A freely available web‐based software that provides users with data on genomic variants, individual research participants' clinical findings, summary‐level genotype and phenotype data across research cohorts, drug structures, and clinical trial outcomes	https://www.cafevariome.org	Lancaster *et al.,* 2015
GenomeConnect	A patient portal providing an opportunity for patients to add their genetic and health information. Data can be matched with queries from clinicians, laboratory personnel and researchers to better interpret the results of genetic testing and build a foundation to support genomic medicine	https://www.genomeconnect.org/	Kirkpatrick *et al.,* 2015
Genetic Alliance	A biobank and repository with stored clinical data and biological samples. Biological samples are annotated with clinical data including disease progression, treatment information, demographic information, lifestyle information and family history. The site is integrated with PEER, a platform that allows patients to share their patient‐reported outcomes, health records and genetic data	https://geneticalliance.org/	Terry *et al*., 2011
MatchMaker Exchange	This exchange allows for genomic discovery through the exchange of phenotypic and genotypic profiles. The platform allows matching of unsolved cases with similar phenotypic and genotypic profiles (matchmaking) through standardized application programming interfaces	https://www.matchmakerexchange.org/	Philippakis *et al*., 2015
Clinical interpretation of variants			
ClinGen	ClinGen is a central resource that defines the clinical relevance of genes and variants for use in precision medicine and research	https://clinicalgenome.org/	Rehm *et al.,* 2015
ClinVar	ClinVar is an archival database that aggregates information about genomic variation and its relationship to human health. ClinVar processes submissions reporting variants found in patient samples, assertions made regarding their clinical significance and other supporting data. The alleles described in submissions are mapped to reference sequences	https://www.ncbi.nlm.nih.gov/clinvar/	Landrum *et al*., 2014
LOVD	An open‐source variation database for the viewing of genomic variants in locus‐specific databases, with phenotype collection. Contains all published variants, including variants of unknown significance	https://www.lovd.nl/3.0/home	Fokkema *et al.,* 2011
Gene2phenotype	The G2P web application provides users with datasets that formalize collections of locus–genotype–mechanism–disease–evidence threads curated from the literature and found to be implicated in the cause of a specific disease or clinical presentation. Furthermore, users can search allele frequency data sourced from public datasets together with the predicted mutational consequence annotations to produce a list of potentially causative genotype(s)	https://www.ebi.ac.uk/gene2phenotype	Thormann *et al.,* 2019
Genome and DNA databases			
Ensembl	Ensembl is a genome browser for vertebrate genomes that supports research in comparative genomics, evolution, sequence variation and transcriptional regulation. Ensembl annotate genes, computes multiple alignments, predicts regulatory function and collects disease data	https://www.ensembl.org	Howe *et al*., 2021
UCSC Genome Browser	An interactive website offering access to genome sequence data from a variety of vertebrate and invertebrate species and major model organisms, integrated with a large collection of aligned annotations. The browser is an open‐source, web‐based tool suite for rapid visualization, examination and querying of data at many levels	https://genome.ucsc.edu	Karolchik *et al*., 2002
gnomAD	This database provides users with both exome and genome sequencing data from a wide variety of large‐scale sequencing projects and makes summary data available for the wider scientific community	https://gnomad.broadinstitute.org/	Karczewski *et al.,* 2020
NCBI RefSeq	RefSeq sequences form a foundation for medical, functional and diversity studies. They provide a stable reference for genome annotation, gene identification and characterization, mutation and polymorphism analysis (especially RefSeqGene records), expression studies, and comparative analyses of DNA. Annotations include coding regions, conserved domains, transcriptional RNAs, sequence tagged sites, variation, references, gene and protein product names, and database cross‐references	https://www.ncbi.nlm.nih.gov/refseq/	Pruitt *et al*., 2006

DECIPHER, Database of Chromosomal Imbalance and Phenotype in Humans Using Ensembl Resources; ENCODE, Encyclopedia of DNA Elements; GARD, Genetic and Rare Diseases Center; gnomAD, Genome Aggregation Database; LOVD, Leiden Open Variation Database; OMIM, Online Mendelian Inheritance in Man; PEER, Promise for Engaging Everyone Responsibly; UCSC, University of California and Santa Cruz Genome Browser.

^a^ References are supplied in Supplementary Data S1.

Despite the refinements in NGS technologies, the diagnostic yield of exome and genome sequencing is estimated at approximately 30%, leaving the majority of tested individuals undiagnosed.^7^ This shortfall has led to the development of genomic matchmaking, where two or more parties looking for cases with similar phenotypes and variants in the same candidate gene can expedite the gene‐discovery process. Currently, platforms including MyGene2,^8^ and GeneMatcher^9^ allow for submission of data from approved researchers or clinicians (Table 1). In addition, the Database of Chromosomal Imbalance and Phenotype in Humans Using Ensembl Resources shares publicly available patient data on rare diseases, detailing > 45 000 copy number and sequence variants, and including > 150 000 phenotype observations.^10^ Additional sites, including Genome Connect,^11^ GeneticAlliance^12^ and MatchMaker Exchange,^13^ have been created for upload of clinical and genetic information by patients and clinicians for unsolved cases.

When interpreting clinical gene variants from genetic testing, websites such as ClinGen,^14^ ClinVar,^15^ Leiden Open Variation Database^16^ and/or gene2phenotype,^17^ represent databases that report variants found in patient samples and provide assertions regarding their clinical significance. Genome browsers such as Ensembl^18^ and the University of California and Santa Cruz Genome Browser,^19^ can be used as graphical viewers that facilitate inquiry‐driven data mining including gene predictions, mRNA alignments and variation data. The Genome Aggregation Database^20^ allows users to connect to an online database and compare individual data to a population database, allowing the identification of pathogenic variants from among common, benign variations in the human genome. In addition, the use of repositories for analysis of large datasets from molecular, clinical and epidemiology data provides an opportunity to compare data easily, and with the promise of precision medicine it can be used for hypothesis generation in addition to hypothesis testing (Table 2).

**Table 2:** Comparison of available online resources for clinical transcriptomics, proteomics, metabolomics, lipidomics and epigenomics for skin disease.

Online resource	Functionality	Source	References^a^
Data repositories			
NCBI GEO	The NCBI is a public archive for gene expression profiling and RNA methylation profiling. RNA‐sequence datasets allow reproducibility of published studies and facilitates its reuse. The resource distributes microarray, next‐generation sequencing and other forms of high‐throughput functional genomics data	https://www.ncbi.nlm.nih.gov/geo/	Barrett *et al*., 2012
ArrayExpress and BioStudies databases	ArrayExpress is an archive of functional genomics data including gene expression, RNA and protein expression and DNA methylation profiling data. It also provides a knowledge base of gene expression profiles. The BioStudies database was established to deal with multimodal data, combining RNA sequencing, protein expression assays and genotyping	https://www.ebi.ac.uk/arrayexpress/ https://www.ebi.ac.uk/biostudies/	Sarkans *et al*., 2021
Skin Science Foundation Bioinformatics Hub	The hub provides a web platform giving open access to curated transcriptomic data from multiple inflammatory skin diseases. It allows users access to compare expression of ~20 000 single genes across multiple skin diseases and identify disease‐specific signatures. This cross‐comparative approach allows discovery of unique disease biomarkers and classifiers, and provides new insights into disease pathogenesis	https://biohub.skinsciencefoundation.org/	Gilliet & Griffiths, 2020
SRA	The SRA is a bioinformatics database that provides a public repository for DNA sequencing data, especially the #short reads# generated by high‐throughput sequencing, which are typically less than 1000 base pairs in length. The archive is part of the International Nucleotide Sequence Database Collaboration	https://www.ncbi.nlm.nih.gov/sra/	Leinonen *et al*., 2010a
ENA	The ENA is Europe's primary nucleotide‐sequence repository, which is an open, supported platform for the management, sharing and archiving of sequence data. Data can be searched and retrieved. Comprehensive sequence similarity searches against ENA data can be performed.	https://www.ebi.ac.uk/ena/browser/home	Leinonen *et al*., 2010b
Transcriptomics resources			
Human cell atlas	This project defines all human cell types in terms of distinctive molecular profiles (such as gene expression profiles) and connects this information with classic cellular descriptions (such as location and morphology). It provides an open, comprehensive reference map of the molecular state of cells in healthy human tissues	https://www.humancellatlas.org/	Regev *et al*., 2017
GTEx	This project is a public resource of tissue‐specific gene expression and regulation. Samples from 54 nondiseased tissue sites across nearly 1000 individuals, primarily for molecular assays including whole genome and whole‐exome sequencing and RNA sequencing. The GTEx Portal provides open access to data including gene expression, quantitative trait loci and histology images	https://gtexportal.org/	GTEx Consortium, 2013
Proteomics resources			
Human Skinatlas	A resource that combines advanced tissue dissection methods, flow cytometry and proteomics (through mass spectrometry) to describe a spatially resolved quantitative proteomic atlas of different layers and cell types in healthy human skin	https://skin.science/	Dyring‐Andersen *et al*., 2020
ProteomeXchange	A publicly available data sharing resource of mass spectrometry proteomics data from humans (4500 datasets) and other main model organisms. Established from the PRIDE database with the aim of standardizing data submission and dissemination of proteomics data worldwide	http://www.proteomexchange.org/	Vizcaíno *et al*., 2014
PeptideAtlas	A multi‐organism, publicly accessible compendium of peptides identified in a large set of tandem mass spectrometry proteomics experiments. Mass spectrometry output files are collected for human, mouse, yeast and several other organisms and searched using the latest search engines and protein sequences	http://www.peptideatlas.org	Deutsch *et al*., 2008
Metabolomics and lipidomics resources			
METLIN	A web‐based data repository to assist in a broad array of metabolite research and to facilitate metabolite identification through mass analysis. METLIN includes an annotated list of known metabolite structural information that is easily cross‐correlated with its catalogue of high‐resolution mass spectrometry spectra and tandem mass spectrometry spectra	http://metlin.scripps.edu	Smith *et al*., 2005
Human Metabolome Database	An electronic database containing detailed information about small molecule metabolites found in the human body. It is intended to be used for applications in metabolomics, clinical chemistry, biomarker discovery and general education. The database is designed to contain or link three kinds of data including chemical, clinical and molecular biology/biochemistry data	http://www.hmdb.ca	Wishart *et al*., 2009
LMSD	This database encompasses structures and annotations of biologically relevant lipids. Users can search using either text‐based or structure‐based search options. The database allows for a review of the nomenclature of lipids and their metabolites, as well as allowing search options based on lipid classification and structure. Users can also perform cross‐referencing of lipids in the system with lipid‐associated proteins and genes in the similar LIPID MAPS proteome database to align proteomics data	http://www.lipidmaps.org/data/structure	Sud *et al*., 2006
Epigenomics resources			
ENCODE	A repository of up‐to‐date human assays to identify likely functional elements, yielding an output of expression level or accessibility in a human tissue, including skin. ENCODE investigators employ a variety of assays and methods to identify functional elements. Regulatory elements are investigated through DNA hypersensitivity assays, assays of DNA methylation and immunoprecipitation of proteins that interact with DNA and RNA, e.g. modified histones, transcription factors, chromatin regulators and RNA‐binding proteins	https://www.encodeproject.org/	ENCODE Project Consortium *et al*., 2020
Roadmap Epigenomics Project	A resource of catalogued normal candidate regulatory elements built around next‐generation sequencing technologies to map DNA methylation, histone modifications, chromatin accessibility and small RNA transcripts in stem cells and primary *ex vivo* tissues involved in human disease. Data are integrated with multiple functional genomic data including chromatin accessibility, RNA sequencing and gene expression to predict chromatin states	http://www.roadmapepigenomics.org/	Roadmap Epigenomics Consortium *et al*., 2015

ENA, European Nucleotide Archive; ENCODE, Encyclopedia of DNA Elements; GTEx, Genotype‐Tissue Expression; NCBI GEO, National Center for Biotechnology Information Gene Expression Omnibus; LMSD, Lipid Maps Structure Database; PRIDE, PRoteomics IDEntifications; SRA, Sequence Read Archive.

^a^ References are supplied in Supplementary Data S1.

### Transcriptomics

Transcriptomics technologies, such as expression arrays and RNA sequencing, can provide an analysis of differentially expressed genes as a transcriptional response of the genome to various environmental stimuli.^21^ Online resources, such as The Genotype‐Tissue Expression project^22^ and The Human Cell Atlas,^23^ have created platforms that link DNA sequencing and multi‐tissue RNA sequencing across donor samples and all human cell types.

### Proteomics

Assessing proteins at the cellular and tissue level can more accurately define the disease state compared with transcriptomics or genomics alone.^24^ Proteomics can lead to biomarker discovery for disease diagnostics, prognostics, prediction of treatment response and development of novel therapeutic targets. For instance, the Human Skinatlas site^24^ provides a comprehensive proteomic overview for measurement and mapping of ~11 000 functional and structural proteins from healthy skin samples.

### Metabolomics and lipidomics

Metabolomics offers an opportunity not only to assess disease pathogenesis and treatment, but also to understand associations with inflammatory pathways, the pathogenic role of skin metabolites and the gut microbiome, and the downstream effects of environmental factors. Using mass spectrometry (MS)‐based high throughput technologies, individual data can be compared to online repositories such as METLIN,^25^ and The Human Metabolome Database (HMDB).^26^

Lipidomics is a newly emerging discipline studying the cellular lipids on a large‐scale throughput analysis of MS‐based data on platforms such as the HMDB.^26^ Given that the skin barrier is mainly comprised of a lipid‐enriched extracellular matrix including ceramides, cholesterol and free fatty acids, interest in the biological processes of lipid metabolism in dermatological diseases is increasing. Currently, databases such as the LIPID MAPS^®^ Structure Database^27^ provide a consortium of lipids identified through lipid experimentation, computational analysis and relevant lipids manually curated from public sources.

### Epigenomics

Although the human genome is largely preserved in all human cell types, there are considerable changes within the epigenomic landscape that underwrite the downstream expression of genes and biological functions. Currently, databases such as the Encyclopedia of DNA Elements Project^28^ provide core gene sets corresponding to major cell types, mapping of patterns of human transcription factors and their key biological features.^28^ Similarly, the Roadmap Epigenomics Project^29^ was created to support reference epigenome production and to coordinate epigenomics data.

## Conclusion

The shift from clinical examination to integrative data analysis can provide a more comprehensive approach to complex disease management. Furthermore, with the recent introduction of omics technologies at a single‐cell level, which have the capacity to profile gene expression patterns and the interactions between individual cells, understanding of human skin and skin diseases is rapidly responding. The future challenge now lies in the ability of these resources to integrate data across multiple omics fields,^30^ and to translate this information into individualized care for patients. To further improve precision medicine, the integration of various biobanks into a global platform is required, with harmonization in the methods of sample collection, storage, analytical methods used and protocols defined in study designs to secure reproducibility of future research.

## CPD questions

### Learning objective

To demonstrate an understanding of the different omic technologies currently being used for dermatology research and the databases and repositories that are used for analysis of data specific to dermatology.

### Question 1

Which of the following is currently *not* an omics platform used for dermatology research?

(a) Proteomics.

(b) Genomics.

(c) Transcriptomics.

(d) Humeomics.

(e) Metabolomics.

### Question 2

Why are genetic disease connection platforms for patients and researchers such as MyGene2 and GeneMatcher so important in genomics?

(a)They can provide gene mutation data for patients.
(b)The use of next‐generation technologies has a diagnostic yield of only ~30%, thus these platforms can help share similar phenotypes and variants to expedite the gene‐discovery process.
(c)Patients and researchers need a café to discuss genetic diseases.
(d)Scientists need a platform to review known genetic mutations.
(e)The diagnostic yield of next‐generation technologies is almost 100%.

### Question 3

Which of the following is *not* a data repository used for dermatology research?

(a)The NCBI Gene Expression Omnibus.
(b)The ArrayExpress and BioStudies databases.
(c)The Sequence Read Archive.
(d)The European Nucleotide Archive.
(e)OrphaNet.

### Question 4

Which high‐throughput technology is currently being used for the evaluation of lipidomics?

(a)Cell culture.
(b) Genome sequencing.
(c)Mass spectrometry.
(d)Whole‐exome sequencing.
(e)Gene panel testing.

### Question 5

Which new omics technology can provide information on the individual interactions between cells and profile gene expression patterns?

(a)Single‐cell level omics.
(b) Genomics.
(c) Single nucleotide polymorphisms.
(d)Small regulatory RNAs.
(e)Mass spectrometry.

## Instructions for answering questions

This learning activity is freely available online at http://www.wileyhealthlearning.com/ced

Users are encouraged to

- Read the article in print or online, paying particular attention to the learning points and any author conflict of interest disclosures.
- Reflect on the article.
- Register or login online at http://www.wileyhealthlearning.com/ced and answer the CPD questions.
- Complete the required evaluation component of the activity.

Once the test is passed, you will receive a certificate and the learning activity can be added to your RCP CPD diary as a self‐certified entry.

This activity will be available for CPD credit for 2 years following its publication date. At that time, it will be reviewed and potentially updated and extended for an additional period.

## Supporting information


**Data S1** References for tables.
