# Supplementary material for: A clinician's guide to omics resources in dermatology
Source: Clin Exp Dermatol. 2022 Mar 3;47(5):858–66. doi: 10.1111/ced.15117 (PMC9314906; doi:10.1111/ced.15117)
Supplement: Supplementary file 1 — Data S1 References for tables. [file CED-47-858-s001.docx]

**A clinician’s guide to omics resources in dermatology**

Doolan BJ^1^, McGrath JA^1^, Onoufriadis A^1^

^1^St John's Institute of Dermatology, School of Basic and Medical Biosciences, King's College London, London, United Kingdom

__________________________________________________________________________________

**Supplementary Information**

*References for Table 1: ^1-19^*

Amberger JS, Bocchini CA, Scott AF *et al.* OMIM.org: leveraging knowledge across phenotype-gene relationships. *Nucleic Acids Res* 2019; **47**: D1038-D43.

Weinreich SS, Mangon R, Sikkens JJ *et al.* [Orphanet: a European database for rare diseases]. *Ned Tijdschr Geneeskd* 2008; **152**: 518-9.

In: *GeneReviews((R))* (Adam MP, Ardinger HH, Pagon RA et al., eds). Seattle (WA). 1993.

Lewis J, Snyder M, Hyatt-Knorr H. Marking 15 years of the Genetic and Rare Diseases Information Center. *Transl Sci Rare Dis* 2017; **2**: 77-88.

Chong JX, Yu JH, Lorentzen P *et al.* Gene discovery for Mendelian conditions via social networking: de novo variants in KDM1A cause developmental delay and distinctive facial features. *Genet Med* 2016; **18**: 788-95.

Sobreira N, Schiettecatte F, Boehm C *et al.* New tools for Mendelian disease gene identification: PhenoDB variant analysis module; and GeneMatcher, a web-based tool for linking investigators with an interest in the same gene. *Hum Mutat* 2015; **36**: 425-31.

Firth HV, Richards SM, Bevan AP *et al.* DECIPHER: Database of Chromosomal Imbalance and Phenotype in Humans Using Ensembl Resources. *Am J Hum Genet* 2009; **84**: 524-33.

Lancaster O, Beck T, Atlan D *et al.* Cafe Variome: general-purpose software for making genotype-phenotype data discoverable in restricted or open access contexts. *Hum Mutat* 2015; **36**: 957-64.

Kirkpatrick BE, Riggs ER, Azzariti DR *et al.* GenomeConnect: matchmaking between patients, clinical laboratories, and researchers to improve genomic knowledge. *Hum Mutat* 2015; **36**: 974-8.

Terry SF, Horn EJ, Scott J *et al.* Genetic Alliance Registry and BioBank: a novel disease advocacy-driven research solution. *Per Med* 2011; **8**: 207-13.

Philippakis AA, Azzariti DR, Beltran S *et al.* The Matchmaker Exchange: a platform for rare disease gene discovery. *Hum Mutat* 2015; **36**: 915-21.

Rehm HL, Berg JS, Brooks LD *et al.* ClinGen--the Clinical Genome Resource. *N Engl J Med* 2015; **372**: 2235-42.

Landrum MJ, Lee JM, Riley GR *et al.* ClinVar: public archive of relationships among sequence variation and human phenotype. *Nucleic Acids Res* 2014; **42**: D980-5.

Fokkema IF, Taschner PE, Schaafsma GC *et al.* LOVD v.2.0: the next generation in gene variant databases. *Hum Mutat* 2011; **32**: 557-63.

Thormann A, Halachev M, McLaren W *et al.* Flexible and scalable diagnostic filtering of genomic variants using G2P with Ensembl VEP. *Nat Commun* 2019; **10**: 2373.

Howe KL, Achuthan P, Allen J *et al.* Ensembl 2021. *Nucleic Acids Res* 2021; **49**: D884-D91.

Karolchik D, Hinrichs AS, Kent WJ. The UCSC Genome Browser. *Curr Protoc Bioinformatics* 2009; **Chapter 1**: Unit1 4.

Karczewski KJ, Francioli LC, Tiao G *et al.* The mutational constraint spectrum quantified from variation in 141,456 humans. *Nature* 2020; **581**: 434-43.

Pruitt KD, Tatusova T, Maglott DR. NCBI reference sequences (RefSeq): a curated non-redundant sequence database of genomes, transcripts and proteins. *Nucleic Acids Res* 2007; **35**: D61-5.

*References for Table 2: ^20-34^*

Barrett T, Wilhite SE, Ledoux P *et al.* NCBI GEO: archive for functional genomics data sets--update. *Nucleic Acids Res* 2013; **41**: D991-5.

Sarkans U, Fullgrabe A, Ali A *et al.* From ArrayExpress to BioStudies. *Nucleic Acids Res* 2021; **49**: D1502-D6.

Gilliet M, Griffiths CEM. The Skin Science Foundation: Promoting Skin Health through Research. *J Invest Dermatol* 2020; **140**: S189-S90.

Leinonen R, Sugawara H, Shumway M *et al.* The sequence read archive. *Nucleic Acids Res* 2011a; **39**: D19-21.

Leinonen R, Akhtar R, Birney E *et al.* The European Nucleotide Archive. *Nucleic Acids Res* 2011b; **39**: D28-31.

Regev A, Teichmann SA, Lander ES *et al.* The Human Cell Atlas. *Elife* 2017; **6**.

GTEx Consortium. The Genotype-Tissue Expression (GTEx) project. *Nat Genet* 2013; **45**: 580-5.

Dyring-Andersen B, Lovendorf MB, Coscia F *et al.* Spatially and cell-type resolved quantitative proteomic atlas of healthy human skin. *Nat Commun* 2020; **11**: 5587.

Vizcaino JA, Deutsch EW, Wang R *et al.* ProteomeXchange provides globally coordinated proteomics data submission and dissemination. *Nat Biotechnol* 2014; **32**: 223-6.

Deutsch EW, Lam H, Aebersold R. PeptideAtlas: a resource for target selection for emerging targeted proteomics workflows. *EMBO Rep* 2008; **9**: 429-34.

Smith CA, O'Maille G, Want EJ *et al.* METLIN: a metabolite mass spectral database. *Ther Drug Monit* 2005; **27**: 747-51.

Wishart DS, Knox C, Guo AC *et al.* HMDB: a knowledgebase for the human metabolome. *Nucleic Acids Res* 2009; **37**: D603-10.

Sud M, Fahy E, Cotter D *et al.* LMSD: LIPID MAPS structure database. *Nucleic Acids Res* 2007; **35**: D527-32.

ENCODE Project Consortium, Moore JE, Purcaro MJ *et al.* Expanded encyclopaedias of DNA elements in the human and mouse genomes. *Nature* 2020; **583**: 699-710.

Roadmap Epigenomics Consortium, Kundaje A, Meuleman W *et al.* Integrative analysis of 111 reference human epigenomes. *Nature* 2015; **518**: 317-30.
